# Appropriateness and Economic Analysis of Conventional Circulating Biomarkers Assessment in Early Breast Cancer: A Real-World Experience from the E.Pic.A Study

**DOI:** 10.3390/curroncol29020039

**Published:** 2022-01-18

**Authors:** Roberta Maltoni, William Balzi, Tania Rossi, Francesco Fabbri, Sara Bravaccini, Maria Teresa Montella, Ilaria Massa, Lucia Bertoni, Fabio Falcini, Mattia Altini

**Affiliations:** 1 Healthcare Administration, IRCCS, Istituto Romagnolo per lo Studio dei Tumori (IRST) “Dino Amadori”, 47014 Meldola, Italy; william.balzi@irst.emr.it (W.B.); mariateresa.montella@irst.emr.it (M.T.M.); ilaria.massa@irst.emr.it (I.M.); lucia.bertoni@irst.emr.it (L.B.); 2 Biosciences Laboratory, IRCCS, Istituto Romagnolo per lo Studio dei Tumori (IRST) “Dino Amadori”, 47014 Meldola, Italy; tania.rossi@irst.emr.it (T.R.); francesco.fabbri@irst.emr.it (F.F.); sara.bravaccini@irst.emr.it (S.B.); 3 Cancer Prevention Unit, Local Health Authority, 47121 Forlì, Italy; fabio.falcini@auslromagna.it; 4 Romagna Cancer Registry, Romagna Cancer Institute, IRCCS, Istituto Romagnolo per lo Studio dei Tumori (IRST) “Dino Amadori”, 47014 Meldola, Italy; 5 Healthcare Administration, Azienda Unità Sanitaria Locale della Romagna, 48121 Ravenna, Italy; mattia.altini@auslromagna.it

**Keywords:** breast cancer, key performance index (KPI), circulating biomarker, appropriateness, economic resource

## Abstract

The risk of relapse for early breast cancer (BC) patients persists even after decades and to date, no specific and sensitive effective circulating biomarker for recurrence prediction has been identified yet. The international guidelines do not recommend the assessment of the serum tumor markers CEA and CA15-3 in the follow-up of asymptomatic early BC patients. In our institute, IRCCS Istituto Romagnolo per lo Studio dei Tumori (IRST) “Dino Amadori”, as part of the E.Pic.A study, which was designed to assess the economic appropriateness of integrated care pathways in early BC, the use of CEA and CA15-3 as circulating tumor biomarkers in early BC patients was evaluated in 1502 patients one year after surgery, from 2015 to 2018, with an overall expense of EUR 51,764. A total of EUR 47,780 (92%) was used for execution of circulating tumor markers in early BC patients with stage 0, I and II tumors, neglecting the current guidelines and considered inappropriate by our professional board. We found that no patients with stage I BC experienced relapse in the 365 days after surgery, and in any case examination of the circulating markers CEA and CA15-3 was considered crucial for diagnosis of relapse. Our findings suggest that this inadequacy is a low-value area, supporting the reallocation of economic resources for interventions of a higher value for patients.

## 1. Introduction

It is well known that breast cancer (BC) is a heterogeneous disease, from a biological point of view and natural history. A systemic cancer from its diagnosis can arise in a very aggressive or more indolent manner, but the risk of relapse remains for all patients who undergo surgery for BC, even up to 30–40 years after diagnosis [1]. Consequently, the great clinical need emerges for the ability to use blood circulating markers that can guide physicians on a possible disease relapse.

Despite the efforts made, up to now, we do not have a biomarker with an optimal sensitivity and specificity suitable for predicting a patient’s disease relapse. A lot of studies have been performed that investigate circulating biomarkers that are useful to predict disease relapse in patients who underwent surgery for early BC [2,3,4,5,6]. For instance, liquid biopsy has the potential to help manage BC during all stages of disease progression. Circulating Tumor Cells, Extracellular Vesicles, and ctDNA have promise as useful tools in this perspective, describing both spatial and temporal tumor heterogeneity and the sub-clonal evolution of the disease through treatment, and allowing disease and risk of progression to be monitored, aiming at improving personalized medicine [7,8,9]. Unfortunately, pitfalls arise due to biological and technical reasons, and the type of detection of the investigated biological markers. In particular, some of the them are not exclusively expressed by tumor cells, but also by inflammatory cells, and in other cases, the biomarker has suboptimal accuracy [10,11].

Blood tumor biomarkers such as Carcino-Embryonic Antigen (CEA) and Cancer Antigen 15-3 (CA15-3) are not recommended by American Society of Clinical Oncology (ASCO), European Society for Medical Oncology (ESMO) and Italian Association of Medical Oncology (AIOM) guidelines in the follow-up of early BC patients [1,12,13], in asymptomatic patients. AIOM guidelines in the absence of clinical suspect of relapse do not recommended intensive follow-up (in terms of radiological and blood laboratory examination) during follow-up programs after surgery. Some authors reported in the literature that radiological studies can give false-positive results and increase costs [14,15,16,17]. This is also true for serum tumor biomarkers with poor sensitivity and specificity; they should not be recommended as clinical surveillance instruments [18,19,20,21].

In a recently published study, it has been reported that in five Italian regions, the percentage of patients undergoing this evaluation in the first year after BC diagnosis appears to be significantly higher than the 20% benchmark, which was defined as taking into consideration stage IV patients, and other specific conditions in which markers can be indicated [22], systematically neglecting the guidelines.

## 2. Findings from the E.Pic.A Study

In our Institute (IRCCS Istituto Romagnolo per lo Studio dei Tumori (IRST) “Dino Amadori”), the E.Pic.A study, which was specifically designed for BC care, was performed to identify inadequacies in the diagnostic, therapeutic and care pathways with reproducible methods, to evaluate the economic appropriateness of integrated care pathways, to balance the best healthcare possible, and to identify areas of wastage to reallocate the economic resources to high-value activities for patients [23]. The study was approved by the Independent Ethical Committee of the IRST (Reg Sperimentazioni n. 1517, Prot 721/2015; date of approval 17 December 2015). For this purpose, a board of professionals identified seven key performance indexes (KPIs) in the pattern of BC diagnosis and treatment based on the current guidelines from the AIOM [13] and the National Comprehensive Center Network (NCCN) [24]. In this article, preliminary data concerning four KPIs comprised in the E.Pic.A study were shown: KPI-1 (pre-surgery) is defined as the proportion of patients with stage I or II disease who underwent hepatic ultrasound, computed tomography, magnetic resonance imaging, position emission tomography, and bone scan; KPI-2 (post-surgery) includes patients at the same stage who received radiological clinical evaluation within 2 months after breast surgery; KPI-3 (subsequent intervention after mastectomy) is defined as the proportion of patients that were subjected to axillary dissection and/or breast reconstruction within 3 months after mastectomy; KPI-4 (chemotherapy timing) means the proportion of patients that received adjuvant therapy within 60 days after surgery [23]. The KPIs were evaluated in terms of appropriateness and costs, showing that 2798 BC patients received a total of 2156 inappropriate examinations, accounting for EUR 573,510.80.

Based on these findings, we decided to perform further analysis on a cohort of 1502 consecutive BC patients without metastatic disease and other cancers who underwent surgery in the years 2015–2018, assessing the appropriateness of conventional circulating markers (CEA and CA15-3) 365 days after surgery (Table 1).

The professional board considered the assessment of these conventional circulating markers inappropriate in asymptomatic patients who underwent surgery for stage 0, I and II tumors (Table 1) as well as for the other KPIs.

The overall cost for CA15-3 and CEA assessment in the 1502 patients in the 365 days following radical surgery was EUR 51,764 (Table 2). This analysis was possible thanks to the access to administrative data. Table 3 shows the overall costs incurred in marker assessment of BC patients with stage 0, I and II tumors (EUR 47,780).

Based on our findings, 92% (EUR 47,780) of the overall costs for circulating tumor marker execution (EUR 51,764) were spent in an inappropriate manner in BC patients with stage 0, I and II tumors.

Considering that the same patients may have performed marker evaluation not only in the first year after surgery but also in the following 5 years and possibly for 10 years after surgery or for the entire life span, the economic impact could not be negligible, especially if translated on a national scale.

Furthermore, the execution of marker detection causes a great deal of emotional stress on patients due to false positive tests (which inevitably generate the execution of instrumental tests) and false tranquility due to a negative outcome when, instead, a metastatic disease may already be present.

Within the 365 days following surgery, 12 out of 1502 patients experienced tumor relapse. The clinical characteristics of the relapsed patients are reported in Table 4.

Six (50%) of the 12 relapsed patients had a diagnosis of triple negative BC (TNBC). Concerning tumor stage, eight patients (66.7%) and four patients (33.3%) had stage III and stage II BC diagnosis, respectively, but no patients with stage I BC relapsed.

Interestingly, the CA15-3 examination of patient 1 resulted 56.8 KU/L when liver metastasis was diagnosed, whereas the value was 150 KU/L and 45 KU/L before neoadjuvant therapy and after mastectomy, respectively. Patient 3 was diagnosed with axillary relapse after self-examination, with CA15-3 measured after biopsy with a value of 58.5 KU/L. The other relapsed patients displayed CA15-3 values within the normal range (0–33 KU/L), and the diagnosis of relapse was possible thanks to self-examination or other instrumental assessment. For instance, after self-examination, four patients were diagnosed with lymph node relapse (two cases) and skin relapse (two cases). One patient was diagnosed with skin relapse during routine mammary ultrasound, and two patients with positive axilla at surgery performed a basal bone scan with evidence of bone metastasis. Two patients that underwent abdominal ultrasound were diagnosed with liver metastasis, and one patient was diagnosed with brain metastasis after magnetic resonance imaging following symptoms occurrence such as vertigo and vomit.

Hence, despite the small case series, we found that none of the 12 relapsed patients had a diagnosis of tumor recurrence following increased values of CEA and CA15-3 and downstream instrumental exams.

## 3. Conclusions

In our real-world experience, we found that no patients with stage I BC experienced relapse in the 365 days after surgery, and in any case, examination of the circulating markers CEA and CA15-3 was considered crucial for diagnosis of relapse.

Our findings identify an area of low-value use of resources that could be better reallocated to interventions with a higher value for the patient. Hence, sharing these results with physicians is noteworthy to redirect the current clinical practice to an improved compliance with the guidelines drafted by scientific associations.

## Figures and Tables

**Table 1:** Distribution of tumors according to staging.

Stage	Year					%
	2015	2016	2017	2018	Total	
0	22	23	13	40	98	6.5
I	234	196	222	212	864	57.5
IIA	77	73	85	84	319	21.2
IIB	37	20	26	36	119	7.9
IIIA	13	10	15	21	59	3.9
IIIB	1	3	2	5	11	1
IIIC	8	6	11	7	32	2
**Total**	**392**	**331**	**374**	**405**	**1502**	**100**

**Table 2:** Monitoring of CEA and CA15-3 execution in the 365 days following radical surgery.

Year (Patients)	2015 (392)		2016 (331)		2017 (374)		2018 (405)		Total (1502)
Marker type	CA15-3	CEA	CA15-3	CEA	CA15-3	CEA	CA15-3	CEA	CA15-3 or CEA
No. of exams	486	352	464	156	641	158	656	161	3074
Total amount EUR	9234 EUR	3872 EUR	8797 EUR	1709 EUR	12,179 EUR	1738 EUR	12,464 EUR	1771 EUR	51,764 EUR
No. of patients with exams	288	222	263	115	328	126	334	122	1222
% of patients with exams	73	57	79	35	88	34	82	30	81

CEA: Carcino-Embryonic Antigen; CA15-3: Cancer Antigen 15-3; EUR: Euro.

**Table 3:** Monitoring of CEA and CA15-3 assessment in the 365 days following radical surgery, for patients with stage 0, I, IIA and IIB tumors.

Tumor Stage 0									
Year (patients)	2015 (22)		2016 (23)		2017 (13)		2018 (40)		Total (98)
Marker type	CA15-3	CEA	CA15-3	CEA	CA15-3	CEA	CA15-3	CEA	CA15-3 or CEA
No. of exams	12	10	11	7	5	3	16	9	73
Total amount EUR	228 EUR	110 EUR	209 EUR	77 EUR	95 EUR	33 EUR	304 EUR	99 EUR	1155 EUR
No. of patients with exams	9	7	8	5	4	2	10	5	34
% of patients with exams	41	32	35	22	31	15	25	13	35
**Tumor Stage I**									
Year (patients)	2015 (234)		2016 (196)		2017 (222)		2018 (212)		Total (864)
Marker type	CA15-3	CEA	CA15-3	CEA	CA15-3	CEA	CA15-3	CEA	CA15-3 or CEA
No. of exams	316	229	292	99	403	92	359	65	1855
Total amount EUR	6004 EUR	2519 EUR	5542 EUR	1089 EUR	7657 EUR	1012 EUR	6821 EUR	715 EUR	31,359 EUR
No. of patients with exams	189	144	165	71	206	73	189	55	750
% of patient with exams	81	62	84	36	93	33	89	26	87
**Tumor Stage IIA**									
Year (patients)	2015 (77)		2016 (73)		2017 (85)		2018 (84)		Total (319)
Marker type	CA15-3	CEA	CA15-3	CEA	CA15-3	CEA	CA15-3	CEA	CA15-3 or CEA
No. of exams	92	68	104	31	131	39	151	52	668
Total amount EUR	1748 EUR	748 EUR	1976 EUR	341 EUR	2489 EUR	429 EUR	2869 EUR	572 EUR	11,172 EUR
No. of patients with exams	49	44	59	26	69	32	79	37	261
% of patient with exams	64	57	81	36	81	38	94	44	82
**Tumor Stage IIB**									
Year (patients)	2015 (37)		2016 (20)		2017 (26)		2018 (36)		Total (119)
Marker type	CA15-3	CEA	CA15-3	CEA	CA15-3	CEA	CA15-3	CEA	CA15-3 or CEA
No. of exams	50	36	29	5	42	9	58	13	242
Total amount EUR	950 EUR	396 EUR	551 EUR	55 EUR	798 EUR	99 EUR	1102 EUR	143 EUR	4094 EUR
No. of patients with exams	30	23	18	4	23	9	27	10	98
% of patient with exams	81	62	90	20	88	35	75	28	82
**Total amount for stage 0, I, IIA and IIB**									**47,780 EUR**

CEA: Carcino-Embryonic Antigen; CA15-3: Cancer Antigen 15-3; EUR: Euro.

**Table 4:** Clinicopathological features of patients who underwent relapse within the 365 days after surgery. CEA was considered normal with values < 5 µg/L. CA15-3 was considered normal with values < 33 KU/L.

Patient Number	Stage	Age At Surgery	Surgery Type	ER	PgR	Ki67/Mib1	HER2	Relapse Site	CEA (ug/L)	CA15-3 (KU/L)
**1**	IIIA	72	M	20%	10%	20%	0	Liver	NP	56.8
**2**	IIIC	53	Q	0%	0%	67%	0	Axillary lymph node	Normal	Normal
**3**	IIA	79	M	0%	0%	80%	0	Axillary lymph node	NP	58.5
**4**	IIA	64	M	0%	0%	70%	0	Skin	Normal	Normal
**5**	IIIC	49	Q	100%	100%	5%	0	Bones	NP	Normal
**6**	IIIB	87	M	90%	35%	21%	0	Skin	NP	Normal
**7**	IIB	51	M	0%	0%	75%	0	Brain	NP	Normal
**8**	IIIC	72	M	100%	0%	25%	+++	Liver	NP	Normal
**9**	IIB	89	Q	50%	10%	40%	0	Bones	NP	Normal
**10**	IIIC	84	M	0%	0%	30%	0	Axillary extension	NP	Normal
**11**	IIIA	47	M	10%	10%	40%	0	Skin	NP	Normal
**12**	IIIB	80	M	0%	0%	35%	0	Liver	NP	NP

CEA: Carcino-Embryonic Antigen; CA15-3: Cancer Antigen 15-3; ER: estrogen receptor; PgR: progesterone receptor; M: mastectomy; Q: quadrantectomy; +++: positive for HER2; NP: not performed.

## Data Availability

The data presented in this study are available on request from the corresponding author.
